# Impact of Trunk Resistance and Stretching Exercise on Fall-Related Factors in Patients with Parkinson’s Disease: A Randomized Controlled Pilot Study

**DOI:** 10.3390/s20154106

**Published:** 2020-07-23

**Authors:** Changhong Youm, Youkyung Kim, Byungjoo Noh, Myeounggon Lee, Jinhee Kim, Sang-Myung Cheon

**Affiliations:** 1 Department of Health Care and Science, College of Health Sciences, Dong-A University, Saha-gu, Busan 49315, Korea; chyoum@dau.ac.kr; 2 Biomechanics Laboratory, College of Health Sciences, Dong-A University, Saha-gu, Busan 49315, Korea; bar1001@nate.com (Y.K.); freestyle710@naver.com (M.L.); lovepg6112@naver.com (J.K.); 3 Department of Neurology, School of Medicine, Dong-A University, Dongdaesin-dong 3-ga, Seo-gu, Busan 49201, Korea; smcheon@dau.ac.kr

**Keywords:** parkinson’s disease, postural deformity, rigidity, falling

## Abstract

Background: This study aimed to examine the effect of a 12-week progressive trunk resistance and stretching exercise program on fall-related factors in patients with Parkinson’s disease (PD). Methods: A randomized study assessed a progressive trunk resistance and stretching exercise program over a 12-week period. A total of 17 patients with PD participated and were randomly allocated into an exercise group (*n* = 10) or a control group (*n* = 7). Participants in the exercise group completed the exercise program in 60- to 90-min sessions for three days per week. Primary and secondary outcome measures included the trunk mobility scale, functional fitness test, standing balance, and sit-to-walk test. Results: The exercise group showed improvements in functional fitness, trunk mobility, standing balance, and dynamic stability compared with the control group (all *p* < 0.05). The 2.44 m timed up and go test (odds ratio (OR): 0.125) and the 2 min step test (OR: 10.584) of the functional fitness test, and the first-step length (OR: 3.558) and first-toe clearance height (OR: 4.777) of the sit-to-walk test, were different between the groups following the exercise program. Conclusion: This 12-week exercise program improved fall-related factors in patients with PD and may lead to prevention of fall-related injuries.

## 1. Introduction

Parkinson’s disease (PD) is a progressive neurodegenerative disease characterized by motor symptoms such as bradykinesia, rigidity, resting tremor, and postural deformities [1]. These complications are disabling and can limit the activities of daily living [1]. Patients with PD often present with postural deformities, such as camptocormia, antecollis, Pisa syndrome, and scoliosis [2,3], which are known to impact the limbs, neck, and trunk [3]. The increased incidence of postural deformities in these patients reduces head stability and impairs the clarity of the visual and vestibular sensory information used in balance control [4]. These deformities have a major negative impact on the occurrence of falls [5], and occur more frequently in patients with PD than in age-matched controls [6]; furthermore, these complications may disrupt gait and produce pain or discomfort [3].

Previous studies have evaluated exercise-based interventions that specifically focused on trunk deformities in patients with PD. Bartolo et al. [7] and Gandolfi et al. [8] revealed that patients with PD achieved significant improvements in postural control and trunk mobility after a 4-week rehabilitation program. However, these studies were limited to a short-term intervention; therefore, they could not provide a reliable basis on the effects of interventions. In addition, studies have demonstrated that 12-week exercise-based interventions that effectively targeted trunk strength, endurance, and mobility improved quiet-standing balance and gait symmetry in patients with PD [9,10]. However, most of these studies were problematic in that they relied on clinical measures, trunk range of motion (ROM), postural control, and gait symmetry assessments, which may limit the influence of postural deformities on the global motor function of patients with PD, including fall-related factors. Several fall-related factors in patients with PD were previously assessed, including the freezing of gait, cognitive impairment, poor leaning balance, lower limb weakness, and reduced mobility [11,12]. Thus, complex measuring is needed to determine whether the promising results in previous studies correspond to a significant improvement in fall-related factors in patients with PD. This study aimed to examine the effect of a 12-week progressive trunk resistance and stretching exercise program on fall-related factors in patients with PD using infrared cameras. We hypothesized that fall-related factors in these patients would significantly improve after participation in this exercise program in comparison with control patients.

## 2. Materials and Methods

### 2.1. Participants

**Figure 1:**
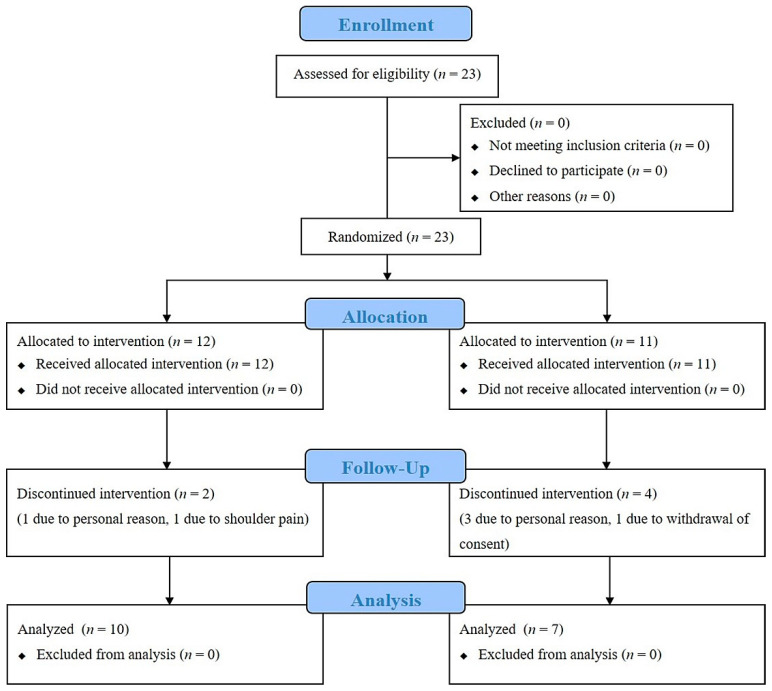
Consolidated Standards of Reporting Trials flow diagram of the study.

This 12-week randomized controlled trial evaluated the effects of a progressive trunk resistance and trunk stretching exercise program on patients with PD. A power analysis based on sample size calculation indicated that we needed 24 participants to determine the effect of a 12-week intervention between the intervention and control condition. A total of 23 participants (one participant withdrew their consent) volunteered to participate, with 12 randomly assigned to the exercise group and 11 randomly assigned to the control group according to a computer-generated randomization sequence. Outcome assessors were trained and blinded to group allocation. Six participants (two in the exercise group and four in the control group) dropped out of the study (Figure 1). Patients with PD who met the United Kingdom PD Society Brain Bank diagnostic criteria were identified [13]. The inclusion criteria for the study were as follows: (a) a diagnosis of idiopathic PD, (b) a Hoehn and Yahr (H&Y) stage of 1 through 3, (c) treatment with dopaminergic medications, and (d) a Mini-Mental State Exam (MMSE) score of greater than 24 points. The study participants did not have a history of orthopedic, neurosurgical, or neurological issues within the preceding six months.

All participants read and signed an informed consent form that was approved by the Dong-A University Institutional Review Board (IRB number: DAUHIRB-16-187). Our study protocol was in accordance with the guidelines of the Declaration of Helsinki and was registered with the Clinical Research Information Service (Korea, https://cris.nih.go.kr; registration number: KCT0003070). This study followed the Consolidated Standards of Reporting Trials (CONSORT) reporting guidelines.

### 2.2. Measurements

Prior to commencing the intervention, participants attended two study-related sessions. In the first session, participants read and signed an informed consent form and underwent assessments of biometric data and MMSE, as well as baseline disease grading using the Unified PD Rating Scale (UPDRS) and the modified H&Y staging system. The second session included functional fitness tests, the trunk mobility scale (TMS) test, a standing balance test, and a sit-to-walk test, which were used to measure the primary and secondary outcomes. Primary and secondary outcome measures were administered at each time point: baseline and 12-week follow-up (immediately after the intervention). All measurements were performed when the participants were in an “on” state and three hours after taking medications to ensure that the full effects of the medications could be realized (see Table S1). The fall-related factors were defined as functional fitness, trunk mobility, standing balance, and dynamic stability [7,8,11,12].

#### 2.2.1. Functional Fitness Test

The functional fitness test consisted of seven assessments that were used to evaluate the functional fitness of patients with PD [14]. Assessments included the 30 s chair stand, arm curl, 2 min step, chair sit-and-reach, back scratch, and 2.44 m timed up and go (TUG) tests.

#### 2.2.2. Trunk Mobility Scale Test

The TMS test, with a between-testers intraclass correlation coefficient of 0.99 and a within-testers consistency of 0.85 [15], was used to evaluate the effects of the intervention on trunk mobility. The TMS results were based on evaluations performed in a static seating position and during six dynamic tests in the frontal, sagittal, and transverse planes [15]. Lower total scores on the TMS reflected higher trunk mobility. 

#### 2.2.3. Standing Balance Test

The standing balance test was performed in a quiet stance using a force plate (OR6-7, AMTI, Watertown, NY, USA). Participants climbed onto the force plate and stood with their big toes and heels together, hands hanging by their sides, knees straight, with their eyes open, and facing forward. This quiet stance was maintained for 40 s with a minimum of disturbances until they were asked to terminate the test [16].

#### 2.2.4. Sit-to-Walk Test

**Figure 2:**
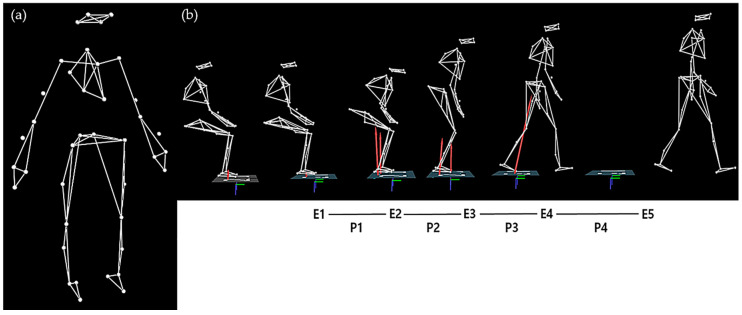
Screenshot of motion capture for the sit-to-walk test. (**a**) A set of 39 retro-reflective markers (14 mm spherical type) were attached to the body using a customized version of the Vicon plug-in gait model; (**b**) Definitions of events and phases. Event 1 (E1) was the point in time when the movement began, and the reflective marker on the C7 vertebra began moving in the anterior direction. Event 2 (E2) was the seat-off point when the anterior–posterior (AP) ground reactive force (GRF) reached its maximum value. Event 3 (E3) was the point when the first step began, and the heel of the foot left the ground. Event 4 (E4) was the moment when the heel of the foot that took the first step contacted the ground. Event 5 (E5) was when the heel of the foot that took the second step contacted the ground [17]. Phase 1 (P1) occurred from E1 to E2, phase 2 (P2) from E2 to E3, phase 3 (P3) from E3 to E4, and phase 4 (P4) from E4 to E5.

The sit-to-walk test was performed using nine infrared cameras (Vicon MX-T10, Oxford Metrics, Oxford, UK) and two force plates. On the basis of a customized version of the plug-in gait marker set, 39 retro-reflective markers (14 mm spherical type) were attached to anatomical landmarks (Figure 2). All markers were secured with double-sided athletic tape to reduce the occurrence of motion artifacts. In order to start the test with maximum speed, participants were seated in a chair with armrests and a back, with the angle of their knee joints set at 90° using a joint angle indicator. The position of their feet was set so that the lower parts of their legs were perpendicular to the ground, and each foot was placed on one of the two force plates. Prior to the test, participants were advised not to use their arms. In addition, when participants began the sit-to-walk test, they kept their hands on their knees in order to minimize the use of their arms.

#### 2.2.5. Progressive Trunk Resistance and Stretching Exercise Program

Participants in the exercise group completed a 12-week progressive trunk resistance and stretching exercise program in 60- to 90-min sessions for three days per week (see Table S2). The control group was advised to continue their daily lives and not to participate in any additional exercise programs or special physical activities.

The exercise program was modified based on previous studies and then reconstructed by an exercise scientist with experience in PD exercise programs. The exercise program consisted of a trunk-specific rehabilitation program in PD [8] and core stability exercise in older adults [18,19]. Exercise program participants were provided with guidance and feedback at all points during the study in order to improve performance. The intensity of exercise was determined based on a 0 to 10 scale for rating of perceived exertion (RPE) [20]. The intensity of the trunk stretching program was calibrated to an RPE of 2 to 4 during weeks 1 through 12. For the resistance program, the intensity was increased every 3 weeks, with an RPE of 2 to 3 for weeks 1 through 3, an RPE of 3 to 4 for weeks 4 through 6, an RPE of 4 to 5 for weeks 7 through 9, and an RPE of 5 to 6 for weeks 10 through 12. The trunk stretching program focused on improving the flexibility of the trunk muscles, including three-dimensional movements. This stretching program targeted muscle groups and isolated muscles, while focusing on the core muscles that connect the upper and lower limbs.

### 2.3. Data Processing

#### 2.3.1. Standing Balance Test

Nexus software (Vicon, UK) was used to collect and analyze the pressure center (COP) data for standing balance. The data sampling frequency was set to 100 Hz and was filtered by a second-order Butterworth low-pass filter with a cutoff frequency of 6 Hz. The analyzed time for standing balance was 30 s, with the deletion of data from the first 5 s after the start signal in order to eliminate initial fluctuations that could have occurred in the experimental environment. The data obtained from a total of three trials were averaged. Analyzed variables included the anterior–posterior (AP) and medial–lateral (ML) velocities and the root mean square (RMS) distances of the COP trajectories in the quiet-stance posture. The AP and ML velocities were calculated separately, with the total distance traveled in the AP and ML directions divided by the analyzed time (*T* = 30 s). The methods of calculation for the AP and ML RMS distances of the COP are shown in Equations (1) and (2) [21,22].

(1) $$RMS_{AP}={\left[\frac{1}{N}\overset{N}{\underset{N=1}{\sum }}{\left(x_{AP\left(n\right)}-\bar{x_{AP}}\right)}^{2}\right]}^{\frac{1}{2}}$$

(2) $$RMS_{ML}={\left[\frac{1}{N}\overset{N}{\underset{N=1}{\sum }}{\left(x_{ML\left(n\right)}-\bar{x_{ML}}\right)}^{2}\right]}^{\frac{1}{2}}$$

#### 2.3.2. Sit-to-Walk Test

Nexus software (Vicon, UK) was also used to collect and analyze the ground reaction force (GRF) and the motion-related data from the sit-to-walk test at maximum speed. The sampling frequencies for motion-related and GRF data were set to 100 and 1000 Hz, respectively. A fourth-order Butterworth low-pass filter was used to filter the motion-related and GRF data with cutoff frequencies of 10 and 25 Hz, respectively. The sit-to-walk test included five events (E1 to E5) and four phases (P1 to P4) (Figure 2). The spatiotemporal variables included the step length, step time, step speed, and toe-clearance height between the first and second steps. The toe-clearance height was calculated to be the maximum vertical position of the marker attached to the second metatarsal bone during the sit-to-walk test.

### 2.4. Statistical Analysis

A Shapiro–Wilk test was used to assess the normality of all data, and an independent-samples *t*-test was used to analyze the differences in demographic characteristics between the exercise and control groups. An analysis of covariance, which was adjusted for age, sex, body mass index, and pre-test scores for all variables of the functional fitness, trunk mobility, standing balance, and sit-to-walk tests, was used to determine the differences in outcome variables between the two groups. Prior to the binary logistic regression analysis, Z-normalization (value—mean/standard deviation) was performed for all tested variables. The binary logistic regression was conducted to determine classifiers for identifying the effect of the exercise program (i.e., pre- and post-test score as dependent variables) in functional fitness, TMS, standing balance test, and sit-to-walk test variables (explanatory variables) within the exercise group, which contained all confounders. IBM SPSS Statistics for Windows, version 21.0 (IBM Corp., Armonk, NY, USA) was used to analyze the data, and the statistical significance level was set at a *p*-value of 0.05.

## 3. Results

Seventeen eligible patients were allocated to either the exercise group or control group (ten in the exercise group and seven in the control group). There were no differences between the demographic or clinical characteristics of the two groups before or after the intervention (Table 1 and Table 2).

Table 3 shows the results for the functional fitness test, trunk mobility scale test, standing balance test, and first- and second-step phases of the sit-to-walk test. After participation in the exercise program, the exercise group showed improvements in the 2 min step test (*p* = 0.018, effect size (ES) = 0.341) and the 2.44 m TUG test (*p* = 0.028, ES = 0.299) of the functional fitness test and in the total TMS score (*p* = 0.023, ES = 0.318) in comparison with the control group. For the standing balance test, the exercise group showed improvements in the AP (*p* = 0.030, ES = 0.293) and ML (*p* = 0.028, ES = 0.299) velocities of the COP trajectory during a quiet stance with their eyes open in comparison with the control group. For the sit-to-walk test, the exercise group showed increases in step length (*p* = 0.003, ES = 0.487) and step velocity (*p* = 0.006, ES = 0.429) during the first-step phase and in step length (*p* = 0.020, ES = 0.332), step velocity (*p* = 0.028, ES = 0.301), and toe-clearance height (*p* = 0.033, ES = 0.285) during the second-step phase in comparison with the control group.

Table 4 summarizes the results of the binary logistic regression analysis for all participants. The binary logistic regression analysis of the effects of the exercise program on baseline and post-intervention measures of the exercise group showed that the odds ratios (ORs) were different for the 2.44 m timed up and go test (OR: 0.125, *p* = 0.034, R_N^2^_ = 0.496) and the 2 min step test (OR: 10.584, *p* = 0.044, R_N^2^_ = 0.451) of the functional fitness test and for the first-step length (OR: 3.558, *p* = 0.030, R_N^2^_ = 0.383) and the first-toe clearance height (OR: 4.777, *p* = 0.045, R_N^2^_ =0.349) of the sit-to-walk test.

## 4. Discussion

This study showed that patients with PD improved their functional fitness, trunk mobility, standing balance, and dynamic stability after a 12-week progressive trunk resistance and stretching exercise program. This study also showed that our measurements could be used to evaluate the effects of the exercise program on patients with PD. Finally, fall-related factors in patients with PD were improved by strengthening of trunk muscle function through our intervention.

Previous studies have mainly focused on the effects of stretching and strengthening exercise on the upper and lower extremity muscles [23,24]. In recent years, some articles have described the rehabilitative effects of exercise programs on trunk posture alterations in patients with PD [7,8,9,10]. These previous studies mainly evaluated the effects of strengthening, stretching, balance, and walking exercises, and found that ROM and posture of the trunk [7], postural control [8], and gait symmetry [9] were improved after these interventions. The present study similarly showed that our intervention improved functional fitness, trunk mobility, standing balance, and dynamic stability, which we considered fall-related factors in patients with PD. Therefore, this exercise program may improve their overall movement. Moreover, our study highlights that exercise programs for these patients are effective when specifically targeted to improving trunk muscle function, which is related to dynamic balance and the central integration of sensory input processes to improve abnormal posture and restricted trunk motion [7,8,9,10].

In addition, this study conducted a logistic regression analysis to determine the effects of our progressive trunk resistance and stretching exercise program. Our measurements were shown to distinguish the effects of the exercise program in patients with PD. While a 4-week trunk-specific exercise program was previously reported to yield a significant reduction in UPDRS 3 scores with mild to moderate motor symptoms [7,8], the findings did not show significant effects on any clinical measures.

Progressive resistance exercises may improve muscle size, muscle strength, muscle endurance, and neuromuscular function and, therefore, may alter activity in the cortex and the basal ganglia and connectivity between and within these regions [25,26,27]. Dopamine deficiency in PD is associated with an increased tonic inhibition of the thalamus and a reduction in the excitatory drive to the motor cortex [27,28]. Additionally, postural deformities in PD patients, which are caused by trunk muscular imbalance, passive element deterioration, and dysfunction of the central nervous system, are related to neuromotor control of the trunk, along with impaired proprioception, poor central nervous system control of body posture, and body schema disorders [3]. In addition, static stretching and proprioceptive neuromuscular facilitation stretching exercises are commonly employed in clinical environments, with the specific aims of increasing joint ROM and reducing the risk of injuries [29]. These interventions may reduce muscle and tendon stiffness and neural adaptations, causing an improved stretch tolerance [30,31,32] and reduction in the spinal reflex excitability of alpha motor neurons through the modulation of I-alpha spinal inhibitory interneurons [8,33]. Thus, our intervention may improve global motor function in these patients, potentially preventing injuries related to falls. We recommend a progressive resistance exercise program as an alternative treatment strategy for the motor symptoms of these patients.

The strength of this study is that it supports the use of a multidisciplinary approach to improving global motor function and preventing falls in PD patients. Furthermore, it confirms the positive effects of progressive trunk resistance and stretching exercise programs. This study also had some limitations, which should be considered. First, this was conducted as a pilot study and had a limited sample size and no follow-up. Therefore, future clinical studies should include more subjects to minimize the dropout effect. Further study with a bigger sample size to confirm obtained trends is warranted. Second, while the PD patients in this study had mild to moderate postural deformities, no stratification of the severity levels of the postural deformities was conducted. Therefore, future studies are needed to consider the effects of similar exercise programs on postural deformities that have different characteristics or different levels of severity. Third, no differences were identified in disease duration, disease severity, or medication dosages between the exercise and control groups. Therefore, these factors could not be considered covariates in the statistical model. Further study is needed to optimize the progressive trunk resistance and stretching exercise program to prevent disability and reduce falling risk in patients with PD. Finally, this study was conducted with nine infrared cameras and two force plates. Although this instrumentation is not a commercial sensor, the infrared camera has an image sensor. In addition, data obtained from the infrared cameras are reliable, as optical motion capture systems are considered the gold standard in motion analysis.

## 5. Conclusions

This study demonstrates that a 12-week progressive trunk resistance and stretching exercise program can improve the functional fitness, trunk mobility, standing balance, and dynamic stability of patients with PD. Additionally, elements of the functional fitness test and sit-to-walk test can be used to evaluate the effects of this exercise program on patients with PD. Therefore, we believe that our intervention can improve fall-related factors in patients with PD, prevent fall-related injuries, and improve the quality of life in this patient population. Our results can be useful as basic data for verification in the field of sensors and are expected to help in developing sensors to quantify trunk rigidity in patients with PD in the future.

## Figures and Tables

**Table 1:** Baseline demographic characteristics of study participants.

	Exercise Group (*n* = 10)	Control Group (*n* = 7)	*t*-Value	*p*-Value
Sex (M/F)	6/4	4/3		
Age (years)	68.0 ± 6.8	72.1 ± 6.0	1.298	0.214
Height (cm)	160.1 ± 8.6	155.8 ± 9.7	0.943	0.360
Weight (kg)	60.2 ± 8.8	62.1 ± 6.3	0.511	0.617
Body mass index (kg/m^2^)	23.5 ± 3.2	25.7 ± 2.6	1.495	0.156
Symptom duration (years)	6.4 ± 3.6	8.0 ± 4.0	1.402	0.181
Treatment duration (years)	3.9 ± 3.1	6.9 ± 3.0	1.971	0.067
L-Dopa equivalent dose (mg/day)	561.0 ± 274.6	852.9 ± 564.4	1.425	0.175

All values are presented as mean ± standard deviations. Abbreviations: M, male; F, female; BMI, body mass index; L-Dopa, levodopa. *t*-values and *p*-values were derived from an independent sample *t*-test.

**Table 2:** Baseline and post-intervention clinical characteristics of study participants.

	Exercise Group		Control Group		*F*-Value	*p*-Value
	Baseline	Post	Baseline	Post		
MMSE (score)	26.60 ± 2.84	26.90 ± 3.73	27.60 ± 1.27	27.29 ± 1.70	0.090	0.914
UPDRS total (score)	64.55 ± 18.33	68.05 ± 18.40	66.00 ± 10.17	62.14 ± 12.51	1.132	0.350
UPDRS III (score)	40.35 ± 10.85	40.05 ± 11.03	44.43 ± 8.80	43.86 ± 7.36	1.677	0.222
H&Y stage	2.40 ± 0.32	2.20 ± 0.82	2.29 ± 0.39	2.29 ± 0.39	0.930	0.418

All values are presented as mean ± standard deviation. Abbreviations: MMSE, Mini-Mental Status Exam; UPDRS, Unified Parkinson’s Disease Rating Scale; H&Y: Hoehn & Yahr. *F*-values and *p*-values were derived from an analysis of covariance (ANCOVA) adjusted for age, sex, body mass index, and pre-test scores.

**Table 3:** Baseline and post-intervention results of the functional fitness test, trunk mobility scale test, standing balance test, and first- and second-step phases of the sit-to-walk test.

		Exercise Group		Control Group		*F*-Value	*p*-Value	ES
		Baseline	Post	Baseline	Post			
**Functional Fitness Test**								
30 s chair sit to stand (reps.)		15.3 ± 6.3	22.0 ± 7.7	13.9 ± 1.7	16.3 ± 4.9	4.460	0.053	0.242
2 min step (steps)		93.9 ± 18.5	113.9 ± 12.0	83.9 ± 15.2	86.7 ± 23.8	7.232	0.018	0.341
2.44 m TUG (s)		8.5 ± 1.8	6.5 ± 1.1	7.5 ± 1.1	7.7 ± 1.1	5.985	0.028	0.299
Arm curl (reps.)	R	28.6 ± 6.2	31.6 ± 4.3	29.4 ± 4.9	29.6 ± 8.2	0.889	0.362	0.060
	L	28.4 ± 5.6	31.3 ± 5.3	27.7 ± 3.2	30.1 ± 6.1	0.097	0.759	0.007
Chair sit & reach (cm)	R	6.0 ± 10.0	8.9 ± 14.5	6.2 ± 16.8	6.1 ± 16.6	0.714	0.412	0.049
	L	5.7 ± 9.8	9.4 ± 16.7	5.9 ± 16.1	5.2 ± 17.7	1.392	0.258	0.090
Back scratch (cm)	R-up	−20.6 ± 9.5	−16.8 ± 11.4	−26.4 ± 16.4	−27.2 ± 15.9	2.443	0.140	0.149
	L-up	−25.0 ± 10.9	−20.9 ± 11.1	−27.8 ± 11.2	−28.3 ± 10.0	3.996	0.065	0.222
**TMS**								
Total score		5.8 ± 2.8	4.2 ± 2.6	6.3 ± 2.6	6.5 ± 2.3	6.520	0.023	0.318
**Standing Balance Test**								
AP velocity (cm/s)		1.37 ± 0.73	0.99 ± 0.32	1.31 ± 0.45	1.35 ± 0.52	5.792	0.030	0.293
AP RMS (cm)		0.64 ± 0.19	0.66 ± 0.21	0.51 ± 0.16	0.52 ± 0.15	0.040	0.844	0.003
ML velocity (cm/s)		1.42 ± 0.59	1.08 ± 0.34	1.35 ± 0.42	1.28 ± 0.34	5.977	0.028	0.299
ML RMS (cm)		0.65 ± 0.17	0.67 ± 0.21	0.56 ± 0.24	0.60 ± 0.26	0.037	0.850	0.003
**First Step Phase for Sit-to-Walk Test**								
Step time (s)		0.40 ± 0.04	0.40 ± 0.03	0.39 ± 0.04	0.41 ± 0.04	1.585	0.229	0.102
Step length (m)		0.51 ± 0.11	0.62 ± 0.04	0.53 ± 0.07	0.54 ± 0.07	13.311	0.003	0.487
Step speed (m/s)		1.31 ± 0.29	1.57 ± 0.21	1.36 ± 0.22	1.32 ± 0.17	10.519	0.006	0.429
Toe clearance height (cm)		5.71 ± 1.10	6.72 ± 0.60	6.11 ± 0.46	5.80 ± 1.81	2.396	0.144	0.146
**Second Step Phase for Sit-to-Walk Test**								
Step time (s)		0.49 ± 0.07	0.46 ± 0.05	0.48 ± 0.05	0.48 ± 0.06	1.520	0.238	0.098
Step length (m)		0.52 ± 0.11	0.59 ± 0.07	0.52 ± 0.10	0.54 ± 0.08	6.953	0.020	0.332
Step speed (m/s)		1.10 ± 0.30	1.29 ± 0.19	1.08 ± 0.19	1.14 ± 0.12	6.027	0.028	0.301
Toe clearance height (cm)		6.89 ± 0.91	7.83 ± 1.10	7.75 ± 1.39	7.36 ± 2.19	5.587	0.033	0.285

All values are presented as mean ± standard deviation. Abbreviations: TUG, timed up and go; reps, repetitions; R, right; L, left; TMS, trunk mobility scale; AP, anterior–posterior; ML, medial–lateral; RMS, root mean square; ES, effect size. *F*-values and *p*-values were derived from an ANCOVA adjusted for age, sex, body mass index, and pre-test scores of the functional fitness test, trunk mobility scale test, standing balance test, and sit-to-walk test.

**Table 4:** Binary logistic regression analysis of the effects of the exercise program within the exercise group.

Variables	Estimate	SE	Odds Ratio	95% CI	*p*-Value	R_N^2^_
**Functional fitness test**						
2.44 m TUG	−2.078	0.981	0.125	0.018–0.856	0.034	0.496
2 min step test	2.359	0.850	10.584	1.065–105.167	0.044	0.451
**Sit-to-walk test**						
First step length	1.269	0.584	3.558	1.133–11.170	0.030	0.383
First toe clearance height	1.564	0.780	4.777	1.037–22.015	0.045	0.349

Reference is a pre-test. Abbreviations: TUG, timed up and go; SE, standard error; CI, confidence interval. R_N^2^_ is the fit statistic for the Nagelkerke model.

## Supplementary Materials

The following are available online at https://www.mdpi.com/1424-8220/20/15/4106/s1. Table S1: List of levodopa equivalent dose; Table S2: Exercise protocols; Table S3: Raw data.
