# Supplementary material for: Impact of Trunk Resistance and Stretching Exercise on Fall-Related Factors in Patients with Parkinson’s Disease: A Randomized Controlled Pilot Study
Source: Sensors (Basel). 2020 Jul 23;20(15):4106. doi: 10.3390/s20154106 (PMC7435366; doi:10.3390/s20154106)
Supplement: Supplementary file 1 [file sensors-20-04106-s001.zip › Supplementary Materials 2. IRB number.pdf]

심의결과 통지서

우 49201 부산광역시 서구 대신공원로 26 ☎051-240-2572,2577 전송 : 051-231-2140

수신	의뢰기관	없음			
	연구책임자	소속	신경과	성명·직위	천상명 · 부교수

과제번호(IRB No.)	DAUHIRB-16-187			
연구 과제명	국문	운동프로그램이 파킨슨환자의 움직임과 안정성에 미치는 영향		
	영문	The effects of trunk exercise program on the mobility and stability in people with parkinson's disease		
연구예정기간	IRB 승인일로부터 ~ 2017년 12월 31일		연구승인유효기간	2017년 09월 26일
심의 구분	●신속심의 ○정규심의		심의 일자	2016년 09월 27일
지속심의주기	○3개월 ○6개월 ●1년 ○기타			
심의결과	●승인 ○시정승인 ○보완 후 신속심의 ○보완 후 정기심의<br>○반려 ○승인된 임상시험의 중지 또는 보류			

심의 의견
수정 및 보완되어야할 사항들이 보완되어 승인합니다.

제출서류 목록
연구계획서요약<br>연구계획서<br>Version : version1.0 2016.08.10. (연구계획서+Version+1.0.hwp)<br>Version : version2.0 2016.09.21 (연구계획서+Version+2.0.hwp)<br>연구계획서 체크리스트<br>연구대상자 설명문 및 동의서<br>Version : version1.0 2016.08.10. (연구대상자용+설명문+및+동의서+Version1.0.hwp)<br>Version : version2.0 2016.09.21 (연구대상자용 설명문 + 동의서 Version 2.0.hwp)<br>연구자용 연구대상자 동의서 체크리스트<br>연구대상자 보상 규약 및 보상에 관한 사항<br>Version : version1.0 2016.08.10. (피해자보상규약+Version+1.0[1].hwp)<br>연구대상자 모집광고 문안<br>Version : version1.0 2016.08.10. (연구대상자 광고문안 Version1.0.pdf)<br>Version : version2.0 2016.09.21 (연구대상자 광고문안 Version2.0.pdf)<br>Delegation Log<br>Version : version1.0 2016.08.10. (delegation log 2010.08.10.pdf)

연구책임자 이력서 전자서식

* 본 임상연구심의위원회는 국제표준화추진위원회(ICH), 약사법, 의료기기법, 생명윤리 및 안전에 관한 법률 등 관련 법규를 준수합니다.

* 본 연구과 이해상충관계가 있는 위원이 있을 경우 해당 위원은 연구의 심의에서 배제하였습니다.

* 심의결과가 시정승인, 보완(신속심의,보완(정기심의)인 경우 보완심의신청서를 제출하시면 신속 또는 정기심의를 거쳐 최종승인을 받을 수 있습니다.

* 심의결과에 이의가 있을 경우 이의신청서를 작성하여 제출하여 주시기 바랍니다.

2016년 09월 27일

동아대학교병원 임상연구심의위원회 위원장

연구자의 책임과 의무
1. 국제표준화 추진회의(ICH), 의약사법, 의료기기법, 생명윤리 및 안전에 관한 법률 등 관련 법규를 준수하여야 합니다.<br>2. 계획서의 승인 이전에 연구 대상자를 해당 임상연구에 참여시키는 것을 금지합니다.<br>3. 연구 대상자에게 발생한 즉각적 위험 요소의 제거가 필요한 경우를 제외하고는 변경계획서에 대한 승인이 내려지기 이전에 원 계획서와 다르게 임상시험을 실시하는 것을 금지합니다.<br>4. 계획서변동/위반이 연구대상자의 안전과 복지나 연구 자료의 아전성에 악영향을 미칠 수 있을 때는 연구자는 이를 인지한지 7일 이내에 위원회에 보고하여야 합니다.<br>**-변동**(Deviation)은 승인된 연구계획과는 다르게 진행되었지만 환자의 권리, 안전, 복지나 연구의 완전성에 직접적인 영향을 끼치지 않는 경우를 말합니다.<br>**-위반**(Violation)은 승인된 연구계획과는 다르게 진행되어 환자의 권리, 안전, 복지, 연구 및 결과 자료에 직접적인 영향을 끼칠 수 있는 경우를 말합니다.<br>5. 위원회의 승인을 받은 연구 대상자 동의서를 사용하여야 합니다.<br>: 연구대상자 설명문 및 동의서가 변경된 경우 이미 진행된 연구대상자에게도 변경된 연구대상자 설명문 및 동의서를 받으시기 바랍니다.<br>6. 모국어가 한국어가 아닌 연구 대상자들에게는 승인된 동의서를 연구 대상자의 모국어로 인증된 번역본을 사용할 것이며, 이러한 동의서 번역본은 반드시 위원회 승인을 받아야 합니다.<br>7. 연구진행에 있어 연구 대상자를 보호하기 위해 불가피한 경우를 제외하고 연구의 어떠한 변경이든 위원회의 사전 승인을 받고 수행할 것 연구 대상자들의 보호를 위해 취해진 어떠한 응급상황에서의 변경도 즉각 위원회에 보고하여야 합니다.<br>8. 연구 대상자에게 중대한 이상반응/의료기기 반응이 발생하였을 경우에는 위원회에 서면으로 보고하여야 합니다.<br>9. 임상시험 또는 연구 대상자의 안전에 대해 유해한 영향을 미칠 수 있는 어떠한 새로운 정보도 즉각적으로 위원회에 보고하여야 합니다.<br>10. 위원회의 요구가 있을 때에는 연구의 진행과 관련된 보고를 위원회에 제출하여야 합니다.<br>11. 연구 대상자 모집광고는 사용 전에 위원회로부터 승인을 획득하여야 합니다.<br>12. 강제 혹은 부당한 영향이 없는 상태에서 충분한 설명에 근거한 동의 과정을 수행할 것이며, 잠재적인 연구 대상자에 게 연구에의 참여여부를 고려할 수 있도록 충분히 기회를 제공하여야 합니다.<br>13. **위원회의 승인은 1년을 초과할 수 없습니다.** 따라서 위원회에서 요구한 지속심의 주기에 따라 지속심의를 신청하여야 합니다.<br>14. 연구가 종료된 경우 종료보고서를 제출하여야 합니다.<br>15. 연구를 조기종료 또는 일시중지 시켰을 경우 위원회에 즉시 일시중지보고서를 제출하여야 합니다.<br>16. 의뢰자는 시험기관의 장과 문서로서 임상시험계약을 체결하여야 합니다.<br>17. 임상시험 실시기관명은 해당 실시기관의 사전 서면동의 없이는 광고 등의 목적에 사용할 수 없습니다.<br>**18. 헬싱키 선언(제19조)에 따라 모든 임상시험은 첫 연구대상자 모집하기 전 공개적으로 접근이 가능한 임상연구등록시스템에 등록하여 이를 공개하여야 하며, 예를 들어, 질병관리본부에서 운영하는 임상연구정보서비스(CRIS, http:// cris.cdc.go.kr)를 이용하실 수 있습니다.**
