# Supplementary material for: Impact of Trunk Resistance and Stretching Exercise on Fall-Related Factors in Patients with Parkinson’s Disease: A Randomized Controlled Pilot Study
Source: Sensors (Basel). 2020 Jul 23;20(15):4106. doi: 10.3390/s20154106 (PMC7435366; doi:10.3390/s20154106)
Supplement: Supplementary file 1 [file sensors-20-04106-s001.zip › Table S2. Exercise protocols.docx]

**Appendix C. Progressive trunk resistance and stretching exercise program**

| **Weeks 1~3** | | | | | | | |
| --- | --- | --- | --- | --- | --- | --- | --- |
|  | Exercise | Position | Reps or time | Set | Rest | Load | RPE |
| Warm-up  (5∼10 min) | Neck joint mobility | Sitting | 10 reps | 1 | - | - | 1 |
|  | Shoulder joint mobility |  |  |  |  |  |  |
|  | Knee joint mobility |  |  |  |  |  |  |
|  | Ankle joint mobility |  |  |  |  |  |  |
|  | Walking with arm swing | Standing | 30 sec | 3 | 30 sec | BW | 2∼3 |
| Trunk dynamic stretching  (15∼20 min) | Upper trunk flexion & extension | Sitting | 20 reps | 2 | 30 sec | - | 2∼4 |
|  | Trunk lateral inclination |  | 20 reps  (R/L) |  |  |  |  |
|  | Trunk rotation |  |  |  |  |  |  |
|  | Trunk side bend |  |  |  |  |  |  |
| Trunk strength  (30∼45 min) | Deadlift | Standing | 10∼12 reps | 2 | 1∼2 min | BW | 2∼3 |
|  | Squat & shoulder press  (chair rise) |  |  |  |  |  |  |
|  | R/L alternating reverse lunge (assist) |  | 10∼16 reps |  |  |  |  |
|  | Modified plank | Supine | 10∼15 sec |  |  |  |  |
|  | Bird dog | Prone | 10∼12 reps |  |  |  |  |
|  | Hip bridge |  |  |  |  |  |  |
| Trunk and lower extremity static stretching  (10∼15 min) | Hamstring stretching | Supine | 30 sec  (R/L) | 2 | - | - | 1∼2 |
|  | Thoracic rotation |  |  |  |  |  |  |
|  | Pelvis rotation |  |  |  |  |  |  |
|  | Hip external rotator & hip extensor stretch  (crossed leg) |  |  |  |  |  |  |
|  | Quadriceps stretching | Standing | 10 sec  3 reps  (R/L) |  |  |  |  |

BW: body weight, R: right, L: left, RPE: rating of perceived exertion

| **Weeks 4~6** |  |  |  |  |  |  |  |
| --- | --- | --- | --- | --- | --- | --- | --- |
|  | Exercise | Position | Reps or time | Set | Rest | Load | RPE |
| Warm-up  (5∼10 min) | Neck joint mobility | Sitting | 10 reps | 1 | - | - | 1 |
|  | Shoulder joint mobility |  |  |  |  |  |  |
|  | Knee joint mobility |  |  |  |  |  |  |
|  | Ankle joint mobility |  |  |  |  |  |  |
|  | Walking with arm swing | Standing | 30 sec | 3 | 30 sec | BW | 2∼3 |
| Trunk dynamic stretching  (15∼20 min) | Upper trunk flexion & extension | Sitting | 20 reps | 2 | 30 sec | - | 2∼4 |
|  | Trunk lateral inclination |  | 20 reps  (R/L) |  |  |  |  |
|  | Trunk rotation |  |  |  |  |  |  |
|  | Trunk side bend |  |  |  |  |  |  |
| Trunk strength  (30∼45 min) | Deadlift | Standing | 12∼15 reps | 2 | 1∼2 min | BW | 3∼4 |
|  | Squat & shoulder press |  |  |  |  |  |  |
|  | R/L alternating reverse lunge |  | 10∼16 reps |  |  |  |  |
|  | Modified plank | Supine | 15∼30 sec |  |  |  |  |
|  | Bird dog | Prone | 10∼15 reps |  |  |  |  |
|  | Hip bridge |  |  |  |  |  |  |
| Trunk and lower extremity static stretching  (10∼15 min) | Hamstring stretching | Supine | 30 sec  (R/L) | 2 | - | - | 1∼2 |
|  | Thoracic rotation |  |  |  |  |  |  |
|  | Pelvis rotation |  |  |  |  |  |  |
|  | Hip external rotator &  hip extensor stretch  (crossed leg) |  |  |  |  |  |  |
|  | Quadriceps stretching | Standing | 10 sec  3 reps  (R/L) |  |  |  |  |

BW: body weight, R: right, L: left, RPE: rating of perceived exertion

| **Weeks 7~9** |  |  |  |  |  |  |  |
| --- | --- | --- | --- | --- | --- | --- | --- |
|  | Exercise | Position | Reps or time | Set | Rest | Load | RPE |
| Warm-up  (5∼10 min) | Neck joint mobility | Sitting | 10 reps | 1 | - | - | 1 |
|  | Shoulder joint mobility |  |  |  |  |  |  |
|  | Knee joint mobility |  |  |  |  |  |  |
|  | Ankle joint mobility |  |  |  |  |  |  |
|  | Walking with arm swing | Standing | 30 sec | 3 | 30 sec | BW | 2∼3 |
| Trunk dynamic stretching  (15∼20 min) | Upper trunk flexion & extension | Sitting | 20 reps | 2 | 30 sec | - | 2∼4 |
|  | Trunk lateral inclination |  | 20 reps  (R/L) |  |  |  |  |
|  | Trunk rotation |  |  |  |  |  |  |
|  | Trunk side bend |  |  |  |  |  |  |
| Trunk strength  (30∼45 min) | Deadlift | Standing | 10∼15 reps | 2 | 1∼2  min | DB  1∼2 kg | 4∼5 |
|  | Squat & shoulder press |  |  |  |  |  |  |
|  | R/L alternating lunge & twist |  | 10∼16 reps |  |  | BW |  |
|  | Plank | Supine | 10∼15 sec |  |  |  |  |
|  | Bird dog  (3sec isometric) | Prone | 10∼15 reps |  |  |  |  |
|  | Hip bridge  (3sec isometric) |  |  |  |  |  |  |
| Trunk and lower extremity static stretching  (10∼15 min) | Hamstring stretching | Supine | 30 sec  (R/L) | 2 | - | - | 1∼2 |
|  | Thoracic rotation |  |  |  |  |  |  |
|  | Pelvis rotation |  |  |  |  |  |  |
|  | Hip external rotator & hip extensor stretch  (crossed leg) |  |  |  |  |  |  |
|  | Quadriceps stretching | Standing | 10 sec  3 reps  (R/L) |  |  |  |  |

BW: body weight, R: right, L: left, DB: dumbbell, RPE: rating of perceived exertion

| **Weeks 10~12** | | | | | | | |
| --- | --- | --- | --- | --- | --- | --- | --- |
|  | Exercise | Position | Reps or time | Set | Rest | Load | RPE |
| Warm up  (5∼10 min) | Neck joint mobility | Sitting | 10 reps | 1 | - | - | 1 |
|  | Shoulder joint mobility |  |  |  |  |  |  |
|  | Knee joint mobility |  |  |  |  |  |  |
|  | Ankle joint mobility |  |  |  |  |  |  |
|  | Walking with arm swing | Standing | 30 sec | 3 | 30 sec | BW | 2∼3 |
| Trunk dynamic stretching  (15∼20 min) | Upper trunk flexion & extension | Sitting | 20 reps | 2 | 30 sec | - | 2∼4 |
|  | Trunk lateral inclination |  | 20 reps  (R/L) |  |  |  |  |
|  | Trunk rotation |  |  |  |  |  |  |
|  | Trunk side bend |  |  |  |  |  |  |
| Trunk strength  (30∼45 min) | Deadlift | Standing | 10∼15 reps | 2 | 1∼2  min | DB  2∼3 kg | 5∼6 |
|  | Squat & shoulder press |  |  |  |  |  |  |
|  | R/L alternate lunge & twist |  | 16∼20 reps |  |  | BW |  |
|  | Plank | Supine | 15∼30 sec |  |  |  |  |
|  | Bird dog  (3sec isometric) | Prone | 15∼20 reps |  |  |  |  |
|  | Hip bridge  (3sec isometric) |  |  |  |  |  |  |
| Trunk and lower extremity static stretching  (10∼15 min) | Hamstring stretching | Supine | 30 sec  (R/L) | 2 | - | - | 1∼2 |
|  | Thoracic rotation |  |  |  |  |  |  |
|  | Pelvis rotation |  |  |  |  |  |  |
|  | Hip external rotator & hip extensor stretch  (crossed leg) |  |  |  |  |  |  |
|  | Quadriceps stretching | Standing | 10 sec  3 reps  (R/L) |  |  |  |  |

BW: body weight, R: right, L: left, DB: dumbbell, RPE: rating of perceived exertion
